# The Treatment of Some of the Forms of Valvular Disease of the Heart

**Published:** 1887-03

**Authors:** 


*The Treatment of some of the Forms of Valvular Disease of the Heart.* By ARTHUR ERNEST SANSOM, M.D. Lond., F.R.C.P., Physician to the London Hospital, &c., &c. Second Edition, with Additions and Corrections. London: J. and A. CHURCHILL. 1886.

The first edition of this book being out of print, after a lapse of more than three years, Dr. Sansom has issued a second edition, and has taken the opportunity to incorporate into the reissue many facts, pathological and therapeutical, which have been made known in the interval. Although the running title of the book is *Valvular Disease of the Heart*, it consists of three sections only, treating respectively of Endocarditis, Mitral Regurgitation, and Mitral Stenosis, lesions of the aortic valve not being considered.

Endocarditis Dr. Sansom divides into four varieties; viz., exudative, sclerous or fibrotic, atheromatous, and ulcerative. In simple endocarditis, attention is called to its frequent development, in children especially, without rheumatic or other pyrexia of any sort, the non-febrile form usually leading to the production of mitral stenosis.

The remarks on the ulcerative form recognise the recent researches of Orth, Socin, Osler, and others: these show how constantly the two varieties of staphylococcus pyogenes are to be found in the vegetations, being usually most numerous at that part where the structureless material joins the granulation tissue; and further, that the "ulcerative" process generally supervenes upon a former endocarditis of the simple variety, the breach of epithelial surface existing in already diseased valves affording an easier entrance to the spores of the micrococcus than a valve structurally intact. It is refreshing to meet with statements and views so sound as Dr. Sansom's in these days when "ulcerative endocarditis" is talked about with a vague and bewildering laxity.

In the lecture on mitral regurgitation, Dr. Sansom gives it as his view that apex systolic murmurs, not presumably associated with organic mitral disease, are due to the muscles around the mitral ostium being enfeebled, and therefore do not contract the orifice sufficiently for the valve-curtains to be capable of completely closing it; the determination of the outline of the heart by percussion, he thinks, serves as means for differential diagnosis of these cases from those of organic mitral disease. In the treatment of mitral incompetence, Dr. Sansom considers that digitalis, which may be often taken for years with advantage, is of little use when there is marked tricuspid regurgitation. Of caffein he thinks highly when compensation has failed and dropsy has been developed; convallaria is but seldom of value as a substitute for digitalis; strophanthus is not mentioned; and urethan, as a hypnotic, he considers as a distinct gain to cardiac therapeutics. Of Oertel's method of treatment of commencing failure of compensation (shown by dyspnœa and commencing dropsy)—viz., promotion of vigorous efforts of the cardiac muscle by severe muscular exercise with a highly nitrogenous diet and a very limited quantity of fluid—Dr. Sansom is in favour, but with some restrictions, and in selected cases only.

The chapter on mitral stenosis is admirable, and illustrated by cardiograms and sphygmograms in a highly instructive manner, showing how the diagnosis may be rendered easily certain, even in the absence of a pre-systolic murmur. In treatment, Dr. Sansom thinks digitalis of much less value, and convallaria of much more value, than in mitral incompetence; and caffein probably less efficient a diuretic in the dropsy of the later stages of stenosis than of incompetence of the mitral valve.

Dr. Sansom's book we heartily commend as most worthy and profitable to be read: it is short in length, simple in language, and sound in logic. If there be any other virtue which a book can possess, let it be added to these.

